# Mode of Genetic Inheritance Modifies the Association of Head Circumference and Autism-Related Symptoms: A Cross-Sectional Study

**DOI:** 10.1371/journal.pone.0074940

**Published:** 2013-09-18

**Authors:** Jonathan M. Davis, Jonathon G. Keeney, James M. Sikela, Susan Hepburn

**Affiliations:** 1 Department of Biochemistry and Molecular Genetics & Human Medical Genetics & Neuroscience Programs, University of Colorado Denver Anschutz Medical Campus, Aurora, Colorado, United States of America; 2 Departments of Psychiatry and Pediatrics, University of Colorado Denver Anschutz Medical Campus, Aurora, Colorado, United States of America; The George Washington University, United States of America

## Abstract

**Background:**

Frequently individuals with autism spectrum disorder (ASD) have been noted with a larger head circumference (HC) than their typical developing peers. Biologic hypotheses suggest that an overly rapid brain growth leads to the core symptoms of ASD by impairing connectivity. Literature is divided however where deleterious, protective and null associations of HC with ASD symptoms in individuals with ASD have been found.

**Method:**

Individuals (n = 1,416) from the Autism Genetic Resource Exchange with ASD were examined for associations of HC with ASD like symptoms. Mixed models controlling for sex, age, race/ethnicity, simplex/multiplex status and accounting for correlations between siblings were used. Interactions by simplex/multiplex were explored. Adjustments for height in a sub-population with available data were explored as well.

**Results:**

A Significant interaction term (p = 0.03) suggested that the effect of HC was dependent on whether the individual was simplex or multiplex. In simplex individuals at mean age (8.9 years) 1 cm increase in head circumference was associated with a 24% increase in the odds of a high social diagnostic score from the Autism Diagnostic Interview – Revised (odds ratio  = 1.24, p = 0.01). There was no association in multiplex individuals. Additionally, individuals classified with a non-verbal IQ <70 were 90% simplex and had a significantly increased head circumference (0.7 cm p = 0.03) relative to a mid-range non-verbal IQ group. Interestingly, children classified with a >110 non-verbal IQ also had an increased HC (0.4 cm p = 0.04), relative to a mid-range non-verbal IQ group, and were 90% multiplex. HC effects do not appear to be confounded by height, however, larger samples with height information are needed.

**Conclusion:**

The potential link between brain growth and autism like symptoms is complex and could depend on specific etiologies. Further investigations accounting for a likely mode of inheritance will help identify an ASD subtype related to HC.

## Introduction

Autism Spectrum Disorder (ASD) is a behaviorally defined developmental disability marked by pronounced deficits in reciprocal social interaction, communication, repetitive behaviors and restricted interests. Currently diagnosed through behavioral observation and caregiver report, efforts to identify biomarkers for the condition have been hampered by heterogeneity of symptom expression and contradictory findings regarding biological indices. Given the within-group differences noted across several areas of inquiry, precision in phenotypic delineation within the autism spectrum is an important step in etiological inquiries into the nature and course of this disorder.

Abnormal brain growth remains a promising etiologic factor of ASD and may contribute to the heterogeneity of symptom expression. It has been noted in numerous reports that individuals with ASD (on average) have a larger head size, more rapid brain growth, and increased neuronal cell numbers over their typical developing peers [1]–[6]. Overly rapid brain growth in ASD likely impairs distal coordination in the brain, supporting the hypothesis that ASD is a disorder of connectivity, perhaps exacerbated or brought about by excessive brain growth [7]–[9]. Specifically, this pattern of development may be unique to the frontal lobe where it has been noted to be an overdeveloped dense structure where neurons are substantially increased in number [4]. This excessive development likely leads to an enlarged size but also promotes a diffuse coordination with distal brain regions and excessive connectivity in local regions, thereby directly impairing frontal lobe functioning and higher order abilities [7], [10].

Functional magnetic resonance imagining among individuals with ASD has also provided evidence that brain function is affected by differing growth trajectories in different brain regions that strain distal connectivity. For example, abnormalities in connectivity have been identified during sentence comprehension [9], executive function [11], and motor functioning tasks [12], and importantly in Theory of Mind functioning [13]. Further, connectivity disruption in frontal temporal language areas, along with intact connectivity to posterior visuospatial regions, may have led to a reliance on visual skills for verbal reasoning in individuals with ASD [14].

Head circumference marks brain growth as noted with a strong correlation with brain volume [15]. Theoretically head circumference could then mark symptoms related to connectivity in individuals with ASD. Perhaps head circumference could mark general social deficits specifically because impaired connectivity has been identified with impaired functional language [16], verbal fluency [17], and self and other-reflection in individuals with autism [18]. However, a number of reports find contradictory relationships between head circumference and symptoms of ASD. These findings range from null associations, potential protective associations, to deleterious associations [5], [19]–[21]. Most of these reports range extensively in sample size, geographic regions of recruitment, and in the methods used to determine enrollment status. ASD is an exceptionally heterogeneous condition where the diagnosis and recorded symptoms are affected by biologic and developmental factors, but could be affected by cultural factors as well [22], [23]. Reports suggest symptom differences between the sexes [24]–[26] and between individuals of different race/ethnicities [23], [27] and with individuals with different modes of genetic inheritance [28]–[30]. Specifically, individuals with different modes of inheritance have been shown to have different distributions of Social Response Scores [29], and likely have different genetic etiologies [28], thus a mode of genetic inheritance could modify head circumference associations with autism like social symptoms. It is possible that the conflicting literature could be due to the analysis of subtly different populations where the role of brain growth may have different effects on ASD-like symptoms.

The aim of this report is to examine the complex relationship between head circumference and ASD-like symptoms in a large cohort of individuals assessed via standardized and uniform procedures and measures. We present this analysis using data from the Autism Genetic Resource Exchange (AGRE) including individuals who meet autism criteria based on the Autism Diagnostic Interview -Revised (ADI-R) and the Autism Diagnostic Observation Schedule (ADOS). We hypothesized that increased head circumference would be associated with increased ASD-like social symptoms as increased neuronal density could be a component of increased size but also effect social functioning through decreased distal connectivity.

## Methods

### Population

In this cross-sectional study we tested associations of head circumference with autism symptoms in individuals from AGRE. AGRE is a large ongoing study collecting phenotypic characteristics and genetic material from individuals with ASD and their families from academic centers across the country. AGRE has uniform recruitment and assessment strategies thoroughly described elsewhere [31]. AGRE data files contain de-identified information of numerous individuals with extensive phenotype information including head circumference and autism diagnostic evaluations. These data were accessed with permission and the current pedigree file (the 15th edition released in October of 2012) and accompanying files were downloaded in December of 2012. This research was approved by the Colorado Multiple Institutional Review Board.

Individuals included in this study meet full autism criteria as determined by the ADI-R and autism or spectrum determined by the ADOS. Individuals with any Fragile X indication, or any non-idiopathic autism indication were removed. Four individuals with a head circumference size outlier values (67–70 cms) were removed. Eighteen individuals ranging in age from late 20′s to late 40′s at the physical exam were removed as well do to their outlying age values. Additionally individuals without testable indications were excluded when applicable. Individuals utilized for the IQ outcome analysis were a subset of the larger original analysis set examining ADI-R outcomes. The data set utilized is further described in Table 1.

**Table 1:** Population Characteristics.

	ADI-R N = 1416	SB IQ N = 515
Sex % Male	79.1%	82.5%
Age, min (mean) max	1.7 (8.9) 24.7	1.8 (9.2) 23.5
White %	68.7%	73.2%
Hispanic %	14.6%	17.1%
Other Race/Ethnicity %	16.7%	9.7%
Simplex %	14.8%	23.3%
Head Circumference, min (mean) max	46.7 (54.0) 62.5	48.0 (54.1) 62.0
ADI-R Social Diagnostic Score, min (median) max	10 (24) 30	10 (22) 30
ADI-R Verbal Communication DS, min (median) max	8 (18) 26 (n = 1032)	8 (17) 26 (n = 497)
ADI-R NVerbal Communication DS, min (median) max	7 (13) 14 (n = 384)	7 (11) 14 (n = 18)
ADI-R Repetitive Behaviors DS, min (median) max	3 (6) 12	3 (7) 12
Stanford Binet NVIQ, min (mean) max	−	42 (97.7) 139
Stanford Binet VIQ, min (mean) max	−	43 (89.8) 145

-not available.

AGRE estimates a general mode of genetic inheritance by differentiating simplex and multiplex individuals. Simplex families were defined in AGRE as those with either a single affected child with an unaffected sibling, or one set of affected identical (monozygotic) twins with an unaffected sibling. Multiplex families were defined as those with more than one affected child (except for one set of monozygotic twins, as noted). It has been hypothesized that the mode of genetic inheritance are different between simplex and multiplex families. Simplex individuals were more likely to have a de-novo, rare copy number variation (CNV) [32] and multiplex individuals are thought to have inherited a complex pattern of risk alleles [33] which may affect ASD symptoms differently [28], [30]. Importantly, enrollment criteria for simplex families require that the simplex individual has an unaffected sibling.

Race/ethnicity is recorded in the AGRE database sample via caregiver report. These consisted primarily of non-Hispanic white (69%), and Hispanic (15%). Numerous other (∼10) race/ethnicities are also included but were grouped as other race/ethnicity for ease of interpretation and power for interaction tests. Less than 4% lacked race/ethnicity information and were included as other.

### Measures

#### Autism Diagnostic Observation Schedule (ADOSx

The ADOS is a clinician administered, structured play diagnostic exam designed to evaluate the core symptoms of autism. The ADOS has 5 versions that are administered according to the child’s developmental ability regardless of age. This study uses the ADOS only as an enrollment mechanism by dropping children with a negative autism ADOS indication.

#### Autism Diagnostic Interview – Revised (ADI-R)

The ADI-R is a 2–3 hour parent interview that is administered by a trained clinician. The ADI-R includes specific questions probing the individual’s history and current report of social, communicative and repetitive behaviors. Higher scores on a diagnostic algorithm indicate greater symptom manifestation. ADI-R sub-domains such as age of first word and social diagnostic score have been used in similar quantitative studies [21], [34].

Individuals with ASD in the AGRE database were administered one of three versions of the ADI. The first is the full version released in 1995, the second is the full version released in 2003, and the third is a short form consisting only of the algorithm questions. Although the versions have subtle differences, the algorithm items are stable between all three versions. Since this study uses only the algorithm questions from the ADI-R, differences in versions of the ADI-R will not affect results. Social Diagnostic Score was the primary outcome used in this analysis. Due to the social score distribution it was dichotomized at the median value, 24 and a logistic model modeling the probability of a high score was used. Communicative and repetitive behavior scores were analyzed in follow up analysis.

#### Vineland Adaptive Behavior Scales (VABS)

The VABS is a parent questionnaire that addresses the child’s personal skills. It is widely used to assess adaptive functioning in social, communication, daily living, and motor skills. The VABS Social Score was a primary outcome of interest, with Daily Living Score, and Motor Skills Score used in follow up analyzes. Due to their normal distribution in this population, the adaptive variables were examined as continuous outcomes in linear regression models. Importantly, the VABS scores in the AGRE data base are raw scores not normed for age and contain scores above 100. To account for this, all models using the VABS controlled for age.

#### Stanford Binet Verbal and non-verbal IQ (SB)

The SB is a commonly used, pyschometrically validated measure of intellectual functioning. The SB Verbal (VIQ) and Non-Verbal IQ (NVIQ) measures are used in this analysis. The distribution of scores in the AGRE data set were normal.

#### Head Circumference (HC)

AGRE follows a standard pediatric head circumference measurement procedure, incorporating anxiety-reducing behavioral techniques. Along with behavioral protocols, AGRE utilizes extensive instructions and a photograph demonstrating proper measuring techniques. The head circumference measurement is taken 3 times and the 2 most similar are maintained and averaged to compute a final HC measurement. Raw HC is used as the primary exposure variable in regression analyzes that control for sex and age at time of head circumference measurement. Individuals’ ages varied slightly between the physical exam and the SB evaluation but were highly correlated (R^2^ = 0.96). On average the SB was administered 4 months after the physical exam.

### Statistical Analysis

Social Diagnostic Algorithm Score from the ADI-R, Standardized Social Score from the VABS, and VIQ from the SB were explored to reflect the primary hypothesis that increased head circumference would affect social symptoms. Follow up analysis included NVIQ from the SB, Communicative Diagnostic Score and Repetitive Behaviors Diagnostic Score from the ADI-R, and Daily Living and Motor Skills scores from the VABS. Additionally, differences in mean head circumference were explored as a function of sex, simplex/multiplex status, race/ethnicity, and specific ASD classification from the ADOS.

Linear mixed effects models were used to test associations of HC with continuous, normally distributed outcome variables. Additionally, mean HC comparisons between groups were also conducted in a linear mixed model framework. Generalized Estimating Equations (GEE) were used to model associations of HC with ADI-R Social Diagnostic Algorithm Score dichotomized at the median value, and ADI-R Repetitive Behaviors Diagnostic Algorithm Score dichotomized at the median value. GEE was also used to test head circumference associations between individuals classified as non-verbal vs verbal from the ADI-R. GEE and linear mixed models were used to account for the correlation among siblings based on family ID. A correlation structure that assumed equal correlation among siblings within a family was used in all linear mixed effects (compound symmetry) and GEE (exchangeable) models. Akaike information criterion supported the use of correlation structures to account for violations of independence over simpler modeling strategies.

Covariates included in all models were; age centered at the mean value at time of the physical exam, sex, race/ethnicity and simplex/multiplex status. HC was also centered at the global mean value for interpretation of other covariates (Table 1). Interactions of HC and simplex/multiplex status were explored along with higher order interactions of all combinations of HC, sex, simplex/multiplex and race/ethnicity. In the case of a significant interaction analyses were stratified for ease of interpretation.

Due to conflicting reports regarding associations of head circumference with various indicators, SB and VABS scores were grouped based on their distributions in follow up analysis. Grouping allowed an evaluation of HC with both improved symptoms and worsening symptoms relative to a mid-range group. In particular in the SB NVIQ analysis individuals were grouped based on the distribution of NVIQ. NVIQ was normally distributed and ranged from 42 to 139 with mean of 98 and was grouped into 3 categories based on visual inspection of a histogram. Group 1 included individuals with a 70 or lower score, Group 2 included individuals who scored 71–110, and Group 3 included individuals scoring more than 110. This analysis was also conducted with VIQ and VABS standardized social scores, daily living scores, and standardized motor skills scores. However, only the NVIQ analysis is presented due to lack of significant findings in other analyses. Grouped analysis was conducted in a linear mixed model framework controlling for the mentioned covariates. The proportions of simplex and multiplex children falling into NVIQ groups were compared via the two sided Fisher’s exact test. Finally height was included as a covariate to assess potential confounding effects. R version 2.15.2 (http://cran.r-project.org/) and the nlme [35] and geepack [36] packages for mixed modeling were used. Mean estimates, Odds Ratios (OR), 95% confidence intervals (95%CI), and corresponding p-values are presented.

## Results

### Population

Initially 1,416 individuals were selected for analysis as described further in Table 1. As analyzes progressed, the population decreased in size (n = 515) due to the completeness of assessments and availability of descriptive measures.

### Head Circumference

At mean age (8.9 years) non-Hispanic white females with ASD presented with a mean HC of 53.3 (95%CI: 53.1 53.5) cm. After controlling for race and ethnicity at mean age, males head circumference on average was 0.9 (95%CI: 0.70 1.10 [p<0.001]) cm larger than females. After controlling for sex at mean age, Hispanic individuals had on average 0.3 (95%CI: −0.61 0.01 [p = 0.037]) cm smaller head circumference then non-Hispanic white individuals. At mean age (and after controlling for the mentioned covariates) there was a nearly significant 0.2 cm increased head circumference in individuals classified as simplex (p = 0.17) over multiplex individuals. Head circumference was not different in children with autism classification over children with spectrum classification (p = 0.25).

### Head Circumference and ADI-R Diagnostic Scores and VABS Adaptive Scores

In the full GEE logistic regression model of a high ADI-R social diagnostic algorithm score including a significant interaction term of simplex by HC, females did not differ from males (p = 0.6). Hispanic-white individuals had a nearly significantly increased risk over non-Hispanic whites OR = 1.37 (95%CI: 0.98 1.87 [p = 0.063]). And, individuals classified as other race/ethnicity had an increased risk of high social score over non-Hispanic whites OR = 1.73 (95%CI: 1.28 2.34 [p<0.001]).

The interaction of HC by simplex/multiplex status was significant (p = 0.027). This suggested a different relationship of head circumference with social diagnostic algorithm score depending upon simplex or multiplex status. Further analysis was stratified based on simplex status or multiplex status (Table 2 and 3).

**Table 2:** Odds Ratio of High Social Diagnostic Algorithm Score in Simplex Individuals.

Simplex Individuals	OR	95% CI	p-value
**Head Circumference**	**1.24**	**1.05 1.45**	**0.014** *
Age	0.99	0.89 1.10	0.810
Sex†	0.88	0.36 2.16	0.790
Hispanic‡	1.14	0.53 2.42	0.738
Other race/eth‡	3.22	1.47 7.02	0.003*

Bold highlights HC association.

* Indicates significant association at alpha <0.05.

† Males compared to females.

‡ Verses Non-Hispanic whites.

**Table 3:** Odds Ratio of High Social Diagnostic Algorithm Score in Multiplex Individuals.

Multiplex Individuals	OR	95% CI	p-value
Head Circumference	1.04	0.98 1.11	0.183
Age	1.01	0.96 1.05	0.170
Sex†	0.94	0.71 1.24	0.660
Hispanic‡	1.43	1.00 2.04	0.050*
Other race/eth‡	1.52	1.11 2.10	0.010*

* Indicates significant association at alpha <0.05.

† Males compared to females.

‡ Verses Non-Hispanic whites.

In simplex cases only, at mean age and after controlling for sex and race/ethnicity each 1 cm increase in head circumference was associated with a 23.7% increase in odds of a high social algorithm score OR 1.24 (95%CI: 1.05 1.46 [p = 0.014]). This relationship was not seen in multiplex individuals.

Finally, there was a near association of increased head circumference with increased ADI-R communication diagnostic score in verbal individuals (p = 0.10). There was a significant association of HC between non-verbal individuals and verbal individuals OR 1.09 (95%CI: 1.01 1.18 [p = 0.035]) (Table 4), where after controlling for the aforementioned covariates increased HC was a risk factor for non-verbal classification. Finally, after adjustment, there was no association of HC with increased repetitive behaviors. VABS outcomes were analyzed similarly and there were no indications of a social diagnostic score and head circumference association. Further, there were no associations with head circumference between VABS social score groups or other VABS derived outcomes.

**Table 4:** Odds Ratio of Non-Verbal Classification from the ADI-R.

	OR	95% CI	p-value
**Head Circumference**	**1.09**	**1.01 1.18**	**0.035** *
Age	0.79	0.75 0.84	<0.001
Sex†	0.92	0.67 1.26	0.591
Hispanic‡	0.97	0.67 1.39	0.850
Other race/eth‡	1.54	1.09 2.19	0.016*
Simple vs Multiplex	0.70	0.47 1.05	0.082

Bold highlights HC association.

* Indicates significant association at alpha <0.05.

† Males compared to females.

‡ Verses Non-Hispanic whites.

### Head Circumference Non-Verbal and Verbal IQ

After adjustment for the aforementioned covariates in a linear mixed model simplex individuals had a 17.7 (95%CI: 14.00 21.13 [p<0.001]) point lower NVIQ than multiplex individuals. There was no significant difference between the sexes (p = 0.07), or between Hispanics and non-Hispanic whites (p = 0.11). Other race/ethnicities, on average, had a 6.5 point (95%CI: 1.29 11.74 [p = 0.016]) lower Non-Verbal IQ than non-Hispanic white individuals.

The results regarding VIQ were similar between simplex vs multilplex individuals, where simplex individuals demonstrated poorer intellectual functioning with a mean 15.1 (95%CI: 11.07 19.10 [p<0.001]) point decrease in VIQ. Other race/ethnicity verses non-Hispanic white averaged significantly lower verbal IQ, −10.7 (95%CI: −16.55 −4.78 [p<0.001]) points. Hispanics also had a slightly lower VIQ than non-Hispanic whites, −5.8 (95%CI −10.53 −1.06 [p = 0.017]). There was no difference between the sexes regarding VIQ, and there was no association of head circumference and VIQ.

**Figure 1:**
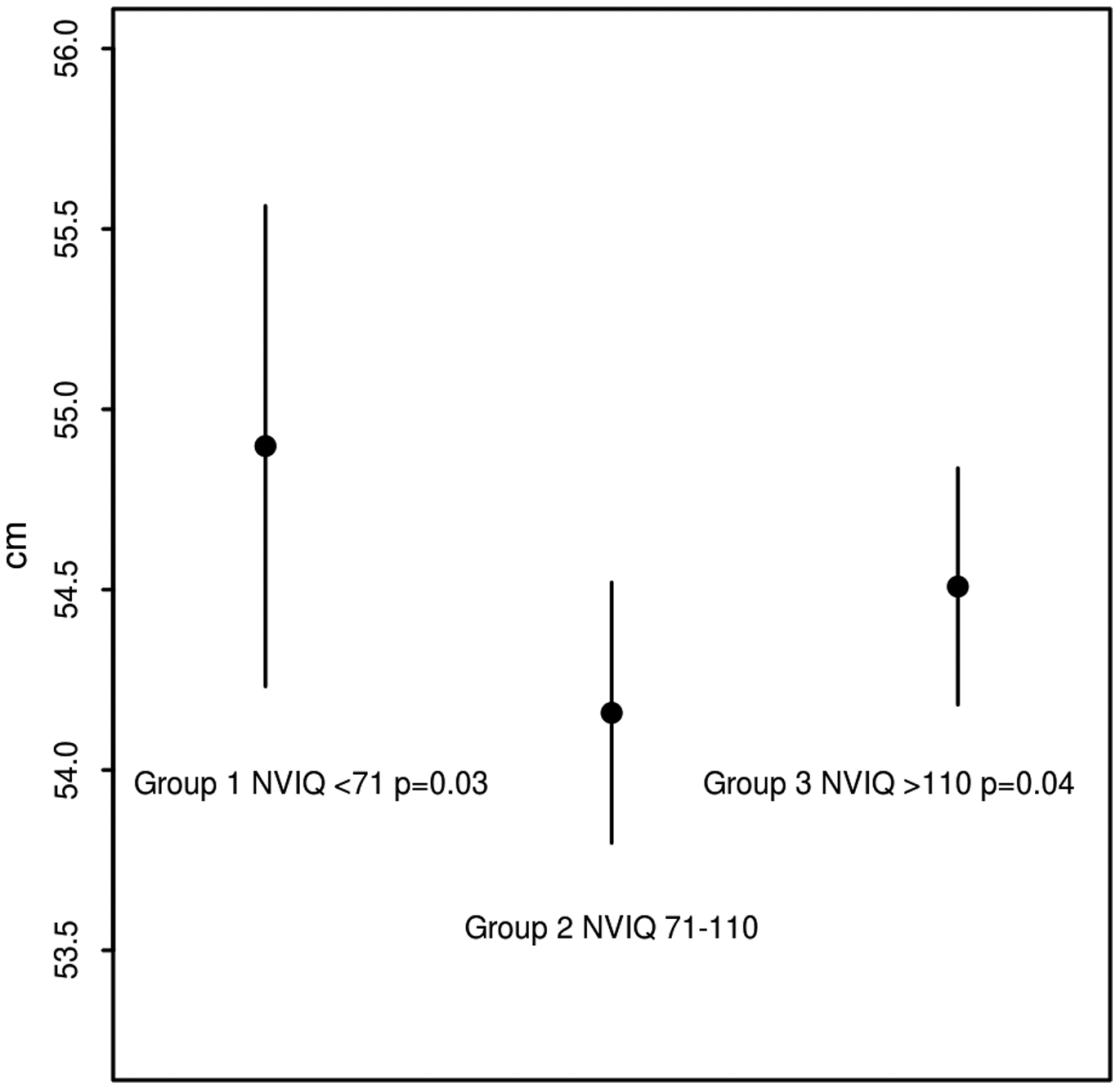
Mean head circumference and 95% CI of NVIQ groups at mean age (9.2 years) in males adjusted for race/ethnicity and simplex status. This pattern was not different in females. Group 1 is >90% simplex individuals, group 2 is 22% simplex, and group 3 is >90% multiplex.

However, an interesting association of head circumference with NVIQ was detected between individuals grouped by their NVIQ values. After controlling for the mentioned covariates in a linear mixed model both group 1 NVIQ < = 70 (cm = 0.74, 95%CI: 0.07 1.41, [p = 0.031]) and group 3 NVIQ > = 110 (cm = 0.35, 95%CI: 0.04 0.66, [p = 0.038]) had significantly increased head circumference on average over individuals classified as group 2 NVIQ = 71–109. (Figure 1).

Follow up analysis tested the proportions of simplex vs multiplex children in NVIQ groups. The mid-range group consisted of 22% simplex and 77% multiplex. Group 1 NVIQ < = 70 consisted almost entirely of simplex children (>90% 28 of 31) and group 3 NVIQ > = 110 was almost entirely multiplex children (>90% 113 of 125), this was significant (over all p<0.0001 Fisher’s exact).

### Height Adjusted Models

Intuitively, height is highly correlated with head circumference even after adjustment for sex and age (p<0.001). However, in the analysis of HC and ADI-R social diagnostic algorithm only 15 simplex individuals had corresponding height measurements and meaningful adjustments were not possible in this small group. Adjustments for height in the analysis of NVIQ groups were likewise limited due to sample size where only 4 individuals were available for analysis in the low nonverbal IQ group. However, 163 (of an original 514) individuals were available for further NVIQ group analysis. Importantly the inclusion of HC in this model did not substantially change the magnitude or direction of the estimates previously detected, (Group 1, 0.5 cm increase p = 0.4, and group 3, 0.4 cm increase p = 0.12). Interestingly Group 3 had a similar estimate and was nearly significantly increased as in the previous unadjusted model. Because the magnitude and direction of the estimates were similar the lack of significance is likely do to power constraints and not confounding effects.

## Discussion

It is well documented that children and adults with ASD have larger head sizes on average than their unaffected peers. The predominant brain growth hypothesis suggests an imbalance in brain growth where accelerated brain growth results in diminished distal connectivity along with localized over connectivity which directly affects ASD symptoms [7], [8], [10], [37], [38]. However, associations of HC with symptoms in individuals with ASD have been contradictory where studies report deleterious, protective and null associations [5], [19]–[21]. Frequently these studies were unable to account for a likely mode of genetic inheritance which could have important effects. Evidence suggests that sex, mode of genetic inheritance, and race/ethnicity could influence symptoms [22], [23], [25], [26], [28]. Importantly, we are able to control for these effects and report a head circumference association with behavioral and cognitive indices that accounts for differences due to race/ethnicity and sex and simplex/multiplex status.

Specifically, we report an increase in ADI-R measured social deficit symptomology related to increased HC only in individuals classified as simplex and not in individuals classified as multiplex. We also report a modest effect where nonverbal individuals had an increased HC over verbal individuals. Finally, we report a paradoxical effect where individuals with lower non-verbal IQ and higher non-verbal IQ had increased head circumference over individuals with a midrange non-verbal IQ. These results are partially consistent with those of Lainhart et al. (2006), who observed an association between macrocephaly (head circumference above the 97th percentile) and both a higher ADI-R social algorithm score and delayed speech in a population of 420 individuals with ASD [21]. These results are also partially consistent with Duetsch & Joseph 2003 who found that individuals with autism maintained a level of non-verbal IQ relative to verbal- IQ with increased head circumference [39]. And the significant interaction also supports the findings of Banach et al (2009) who reported head circumference effects specific to simplex females [25].

A striking difference regarding the proportion of simplex and multiplex individuals in NVIQ groups further suggests different etiologies. The most impaired group had the largest average HC and was predominantly comprised of simplex individuals. At the same time the group with a higher than average NVIQ also had an increased HC and was almost entirely comprised of multiplex individuals. These associations may offer a partial explanation of the contradictory findings in the literature. Perhaps brain growth can affect symptoms differently depending on the specific etiology of ASD. ASD is a common but remarkably heterogeneous condition. Differences in the findings across studies may reflect underlying etiological heterogeneity, as well as behavioral heterogeneity, influenced in part by individual differences in sex, race/ethnicity and a pattern of genetic inheritance.

The diminished distal connectivity is believed to contribute to ASD symptoms. The findings presented support this effect, in part, regarding social symptoms. Further the lack of a HC association with repetitive behaviors also supports a specific symptomology related to HC. Notably social impairment was not directly identified utilizing the VABS however. This could further imply highly specific social like functioning as the VABS and ADI-R are not perfectly correlated instruments and investigations utilizing the Social Response Scale could prove fruitful. These findings also suggest that patterns of genetic inheritance may matter. The classification of multiplex/simplex is challenging, however, as subtle social symptoms observed in family members of simplex individuals may actually be sufficient for classification of a multiplex individual [28] [25]. Our findings would be robust to the misclassification of simplex children as the difference tested would likely underestimate the HC effect in correctly classified groups.

While no significant associations were found in height adjusted models the sample available was greatly diminished, nearly 90% in the first analysis modeling a risk of high social score. Trends remained in direction and size in the second analysis presented. While height and HC are correlated, they are not perfectly so, and this analysis suggests height was not confounding HC associations. Thus a portion of brain growth irregularities in ASD could be independent of body growth. Developmental studies both within humans and across species suggest that body size may have a smaller influence on brain size than originally thought [40]. Examinations of orangutan and gorilla brains indicate that the cellular processes that determine brain size are more conserved than those that control body size, which can diverge substantially [41]. Because brain growth precedes body growth in mammalian development [42], it has been proposed that “body size is not an independent parameter for evaluating quantitative aspects of the human brain” [40]. Although much of the data that supports an uncoupling of brain size from body size evaluated brain size across species, the human brain does not deviate from this trend.

A number of reports have linked brain growth irregularities with ASD, and therefore, candidate genes known to influence brain growth may prove to be informative modifiers of ASD. One such candidate is the DUF1220 protein domain that shows an extreme increase in copy number specifically in the human lineage [43], [44]. DUF1220 copy number (i.e. DUF1220 dosage) has been strongly associated with brain size across primate lineages and in individuals with macrocephaly and microcephaly, as well as in healthy individuals [45]. In addition, large scale copy number variants (CNVs) that flank and include DUF1220 copies have been identified in individuals with ASD, schizophrenia and other neurodevelopmental conditions [46]–[48]. While DUF1220 domain sequences are typically unexamined in genetic disease studies due to their high copy number, future genetic research in ASD may benefit from examination of DUF1220 copy number in the brain growth irregularities associated with ASD.

### Limitations

There are limitations to this study that are important to consider when interpreting results. Importantly this study is cross-sectional and assumes that overly rapid brain growth is marked with increased head size controlled for sex, age, race/ethnicity and simplex/mutiplex status. HC differences are known to be more pronounced at younger age ranges than those presented here, however these results may underestimate HC related effects due to more subdued HC differences over time. Longitudinal investigations that can identify a rate of head growth in a critical early time period in development should increase the precision of these investigations as participants in this study are older. Further, due to caregiver report, the use of the ADI-R may be suboptimal in identifying specific autism related social symptoms related to an enlarged HC. The use of additional tools such as the Social Response Scale will further inform of HC related social behavioral deficits. While height does not appear to confound associations additional information is necessary to fully account for the effects of height.

This study does have offsetting strengths, including its large sample size, broad recruitment geography, highly standardized enrollment criteria and the use of a multivariate analysis accounting for a pattern of genetic inheritance not frequently utilized in similar HC related phenotipic studies.

### Conclusions

ASD is an exceptionally heterogeneous condition the symptoms of which are affected by brain growth as well as other factors. However the effects of brain growth on ASD may be different depending on a pattern of genetic inheritance. Those who may have inherited ASD, as indicated by affected siblings, have a different symptomology related to HC than those who’s ASD may be caused by a de-novo genetic event, as indicated by the presence of unaffected siblings. Further research linking symptoms to HC, such as a HC related ASD subtype, needs to account for a likely pattern of inheritance.
